# *Notes from the Field: *Francisella tularensis Type B Infection from a Fish Hook Injury — Minnesota, 2016

**DOI:** 10.15585/mmwr.mm6607a3

**Published:** 2017-02-24

**Authors:** Tory Whitten, Jenna Bjork, Dave Neitzel, Kirk Smith, Maureen Sullivan, Joni Scheftel

**Affiliations:** ^1^Infectious Disease Epidemiology, Prevention, and Control Division, Minnesota Department of Health; ^2^Public Health Laboratory Division, Minnesota Department of Health.

On June 27, 2016, the Minnesota Department of Health (MDH) Public Health Laboratory (PHL) was notified of a suspected *Francisella tularensis* isolate cultured at a hospital laboratory. The isolate was confirmed as *F. tularensis* type B at MDH PHL by reverse transcription–polymerase chain reaction, culture, and direct fluorescent antibody testing. *Francisella tularensis* subspecies *tularensis* (type A) and *holarctica* (type B) bacteria are the causative agents of tularemia.

MDH initiated an epidemiologic investigation to identify potential exposure sources and risks to additional persons. The case occurred in an immunocompetent white, non-Hispanic woman aged 67 years from Sherburne County, Minnesota. On June 18, she was fishing on a freshwater lake in northeastern South Dakota. While removing a hook from a fish, the hook penetrated the pulp of the patient’s left middle finger. On June 21, she developed pain and swelling at the site of the puncture and was seen at an urgent care center where she received an injection of ceftriaxone and was prescribed oral cephalexin. The pain and swelling did not improve, and she was seen by her primary care provider the next day. Because of concern about a possible joint infection, the patient was referred to an orthopedic specialist, who saw her on June 23, at which time an enlarged, tender left axillary lymph node was noted. The orthopedist drained the finger wound, collected a swab of cloudy, nonpurulent fluid for culture, and changed the patient’s antibiotic to ciprofloxacin. After MDH PHL confirmed tularemia on June 28, the patient was seen again by the orthopedist; by this time she had developed an eschar-like ulcer. An infectious disease consultation was obtained, ciprofloxacin was discontinued, and a treatment course of doxycycline was initiated and ultimately continued for 5 weeks. As of July 8, the lymphadenopathy had resolved and the ulcerated wound was improving. MDH epidemiologists shared exposure information with the South Dakota Department of Health; no other cases of tularemia were reported from this area of South Dakota in 2016 (South Dakota Department of Health, personal communication, January 4, 2017).

Tularemia can cause a wide range of symptoms in humans, depending upon the route of inoculation. The infection is often characterized by fever, lymphadenopathy, and an ulcer at the site of cutaneous inoculation. Type A is frequently associated with lagomorphs (hares, rabbits, and pikas), and type B is frequently associated with rodents and aquatic environments. Type A is often considered more virulent to humans than is type B, but subtypes exist that are associated with varying degrees of severity (*1*,*2*). Exposure routes include animal contact, arthropod (ticks and biting flies) bites, and exposure to natural waters (*1*–*5*). During 1994–2015, 10 tularemia cases were confirmed in Minnesota residents; five were caused by *F. tularensis* type B (MDH, unpublished data, 2009–2015) (*5*).

Although tularemia is rarely diagnosed in Minnesota, there has been one other culture-confirmed case of a tularemia type B wound infection that resulted after lake water exposure of a superficial cut, sustained while shaving (MDH, unpublished data, 2012). Inoculation by fish hook represents a novel exposure to *F. tularensis*. This and the previous Minnesota case highlight the significance of freshwater exposure in cases of tularemia, the importance of obtaining a thorough exposure history, and the importance of obtaining wound cultures, especially when wound infections do not respond to empiric antibiotic therapy. Prompt diagnosis and initiation of appropriate antibiotics, consistent with current practice guidelines, can prevent serious illness in tularemia cases (*6*).
